# Bioprocessing of Grape Pomace for the Development of a Nutraceutical Formulation: Bridging Winemaking By-Products and Functional Innovation

**DOI:** 10.3390/foods14223967

**Published:** 2025-11-19

**Authors:** Simona Piccolella, Lucia Mucci, Francesca Prato, Severina Pacifico

**Affiliations:** Department of Environmental, Biological and Pharmaceutical Sciences and Technologies, University of Campania “Luigi Vanvitelli”, Via Vivaldi 43, 81100 Caserta, Italy; lucia.mucci@studenti.unicampania.it (L.M.); francesca.prato@studenti.unicampania.it (F.P.); severina.pacifico@unicampania.it (S.P.)

**Keywords:** grape pomace, malvidin 3-*O*-glucoside, low-impact extraction, functional jelly candies, in vitro digestion, consumer acceptance

## Abstract

Grape pomace, the main by-product of winemaking, represents a promising source of anthocyanins for sustainable food applications. This study reports their low-impact aqueous extraction, yielding a two-step isolation of malvidin 3-*O*-hexoside (94% purity) characterized by spectroscopic and mass spectrometric analyses. The pure molecule was embedded into a jelly candy to develop a nutraceutical prototype, whose colorimetric analysis revealed a stable red hue. The jelly showed time-dependent release of the anthocyanin and strong antiradical capacity. The in vitro digestion confirmed 37% release in the oral phase, 55% in the gastric phase, and complete degradation in the intestinal phase, offering key insights for developing a further advanced strategy to enhance the bioaccessibility throughout the entire gastrointestinal tract. A consumer test (*n* = 116) indicated good acceptability, particularly among younger and more experienced supplement users. Overall, the study contributes to circular economy strategies towards a more resilient, responsible, and sustainable production system.

## 1. Introduction

The growing demand for natural, functional, and sustainable ingredients in food systems has accelerated research into bioactive compounds derived from agro-industrial by-products [1]. Among these, anthocyanins stand out for their dual role as natural pigments, responsible for the red-to-purple hues of many fruits and vegetables, and for their well-documented antioxidant, anti-inflammatory, and potentially health-promoting effects [2,3]. This functional versatility makes them highly attractive for clean-label food applications [4].

A major challenge in the valorization of anthocyanins lies in achieving an optimal balance between sustainability, extract purity, and technological functionality. Although various studies have focused on anthocyanin-rich extracts obtained from fresh fruits or high-value matrices [5,6], increasing attention is being paid to cost-effective and abundant agri-food residues such as grape pomace [7,8]. Despite its partial valorization, grape pomace remains one of the most widely available winemaking by-products worldwide. Rich in polyphenols and fibers [9], it represents a promising feedstock for integrated, circular bioeconomy strategies [10].

In this context, traditional water-based extraction techniques, such as aqueous hot extraction, warrant renewed attention. Often considered less advanced than modern “green” technologies, such as ultrasound-assisted extraction, pressurized liquids, or supercritical fluids, these conventional methods offer distinct practical advantages: they are solvent-free, cost-effective, easily scalable, and compliant with food-grade regulations. Although decoction typically involves boiling, similar hot-water extractions conducted at sub-boiling temperatures retain many of the same advantages [11,12,13]. Thermal treatment in particular may enhance extraction yields by promoting anthocyanin diffusion from cell walls and complex matrices, an especially relevant factor when the goal is to isolate purified anthocyanins for specific food applications [14].

The selective recovery of pure anthocyanins, as opposed to crude polyphenolic mixtures, offers clear benefits: more accurate dosing, greater chemical stability, enhanced sensory predictability, and improved regulatory compliance. Nevertheless, most studies still rely on anthocyanin-rich extracts, limiting our understanding of how isolated anthocyanins behave as functional food ingredients under real or simulated processing conditions.

In the present study, an aqueous hot extraction, followed by clarification and drying, was employed to isolate a single, chemically characterized anthocyanin from grape pomace. This compound was then incorporated into a simple gelled food matrix composed of agar, fructose, and lemon juice, three food-grade, plant-based, and widely recognizable ingredients, to assess its performance as both a natural colorant and antioxidant.

The choice of a confection-like system (gelled matrix) was driven by multiple considerations: sensory appeal, visual stability, ease of dosing, and widespread consumer familiarity. In particular, the formulation was designed as a low-ingredient, plant-based product suitable for a broad range of potential consumers, including, but not limited to, children. This approach also aligns with consumer preferences for minimal processing, ingredient transparency, and full naturality, factors that play a critical role in market acceptance of functional foods and nutraceuticals. In addition to extraction and application, the study included an evaluation of the anthocyanin in vitro bioaccessibility. A preliminary consumer survey was also conducted to assess sensory acceptance and overall appeal of the final gelled product, offering further insight into its practical potential as a functional food item.

These complementary analyses aim to provide a comprehensive understanding of the compound’s functional potential and safety in food-related applications.

Finally, the study aims to broaden current perspectives on extraction methodology by demonstrating how conventional thermal techniques, when applied thoughtfully to high-volume food waste, can enable sustainable innovation without sacrificing functional performance. The successful use of a low-impact aqueous process to generate an anthocyanin ingredient suitable for food application supports a re-evaluation of “low-tech” processes in modern food chemistry, particularly when optimized for scalability, safety, and environmental compatibility.

## 2. Materials and Methods

### 2.1. By-Products and Waste Management: Survey Design for Wine Industry Stakeholders

A structured survey was developed and distributed in digital format to all the wineries in the Campania region (Italy). The survey was anonymous and designed to be completed in under five minutes. It included both multiple-choice and closed-ended questions divided into two main sections (Figure S1). The first section was aimed at collecting general information to categorize the companies based on location (province) and vineyard size. The second section focused on estimating the average quantities of key by-products (i.e., grape pomace, stalks, and pruning residues) produced per hectare. Moreover, the main destination of these products was asked, choosing among seven categories: distillation, agronomic, zootechnical, industrial, and energy-related.

Data collected were analyzed in an aggregated form, ensuring that individual responses could not be traced back to specific participants.

### 2.2. Materials

The chemicals and reagents used, as well as Amberlite XAD-4, were purchased from Merck Life Science S.r.l. (Milan, Italy), whereas MeOH, EtOH (analytical grade), and formic acid (mass spectrometry grade) were from VWR International (Milan, Italy). Ultrapure water was obtained from a Milli-Q system (Millipore, Bedford, MA, USA). Solvents used for instrumental analyses (mass spectrometry grade) were purchased from Romil Ltd. (Cambridge, UK).

### 2.3. Grape Pomace Collection and Extraction/Fractionation Procedure

Red grape pomace from the ‘Piedirosso’ grapes was collected in October 2024 from a winemaking facility near Benevento (Campania region, Italy) and immediately frozen at −80 °C until use. Then, it was lyophilized for 72 h by the Freeze Dryer FD-1A-50 (Biocool, Beijing, China) and ground to a fine powder. An aliquot (20 g) underwent water extraction at 70 °C for 30 min (1:30 ratio, *w*:*v*), followed by centrifugation at 4800 rpm for 5 min in a Beckman GS-15R centrifuge (Beckman Coulter, Milano, Italy) equipped with an S4180 rotor. The supernatant (Gp-1) underwent gel permeation chromatography (GPC) on Amberlite XAD-4 polystyrene-divinylbenzene resin, eluting with water (Gp-2a), MeOH (Gp-2b), and acidified MeOH (Gp-2c). The latter, obtained with a yield of 2.6% based on the dry weight of the grape pomace, was thoroughly investigated, as described in the following paragraphs.

### 2.4. Spectroscopic Investigation

UV-Vis spectrophotometric analysis was performed using a Cary 100 instrument (Agilent, Milan, Italy) over the 200–800 nm wavelength range, with a blank as reference.

Attenuated Total Reflectance-Fourier Transform InfraRed (ATR-FTIR) analysis was carried out using an IRXross FT-IR spectrophotometer (Shimadzu, Tokyo, Japan). Spectra were acquired in the 4000–500 cm^−1^ range at a resolution of 4 cm^−1^, averaging 45 scans per measurement.

Spectral data were processed using OriginPro 2015 software (OriginLab Corp., Northampton, MA, USA).

### 2.5. HPLC-UV-DAD and UHPLC-HRMS Analysis

The HPLC-UV-DAD analyses were performed by the HPLC 1260 INFINITY II system (Agilent, Santa Clara, CA, USA), equipped with an autosampler (G7129A), a DAD-UV-visible detector (G7115A), and a Quaternary pump (G7111A). The chromatographic separation was achieved on a Kinetex^®^ EVO C18 column (100 × 3 mm, 5 μm; Phenomenex) at 40 °C with an injection volume of 2 µL and using as mobile phase water (solvent A) and acetonitrile (solvent B), both acidified with formic acid (1%). The linear elution gradient started with 2% B and ramped to 98% in 10 min with a flow set at 0.5 mL min^−1^. The total run time was 13 min, considering also the column re-equilibration. The UV-Vis features were monitored at 280 ± 4, 325 ± 4, and 525 ± 4 nm.

High-resolution mass spectra were recorded by the AB SCIEX TripleTOF^®^ 4600 spectrometer (AB Sciex, Concord, ON, Canada), equipped with a DuoSpray™ ion source operating in negative and positive electrospray ion mode, and calibrating the mass-to-charge (*m*/*z*) values in all scan functions through the APCI probe. TOF-MS experiments were combined with MS/MS in Information Dependent Acquisition (IDA) mode, consisting of a full scan TOF survey (accumulation time 250 ms, 270–1500 Da) and eight IDA MS/MS scans (accumulation time 100 ms, 100–1350 Da). Other source and analyzer parameters were the following: curtain gas 35 psi, nebulizer/heated gases 60 psi, ion spray voltage −4.5 (5.5) kV, interface heater temperature 600 °C, declustering potential 80 V, collision Energy (CE) 45 V, CE spread 10 V. The mass spectrometer was coupled to the NEXERA UHPLC system (Shimadzu, Tokyo, Japan). The chromatographic method was set as described before, except for the column that was the Luna^®^ Omega Polar C18 (50 × 2.1 mm i.d., 1.6 μm particle size; Phenomenex, Torrance, CA, USA). The instrument was controlled by Analyst^®^ TF 1.7 software, while data processing was carried out using PeakView^®^ software version 2.2.

### 2.6. Antiradical Capacity Assessment

The antiradical capacity of Gp-2c was assessed towards 2,2-diphenyl-1-picrylhydrazyl (DPPH) radical and ABTS [2,2′-azinobis-(3-ethylbenzothiazolin-6-sulfonic acid)] radical cation, as previously described [15]. Final tested concentrations were 1, 2.5, 12.5, 25, and 50 µg mL^−1^. In both cases, Trolox^®^ (Sigma-Aldrich, Milan, Italy) was used as the positive standard at 2, 4, 8, 16, and 32 µM.

Two independent measurements were carried out, each one in triplicate. All data were expressed as mean ± standard deviation (SD).

### 2.7. Gp-2c Based Jelly Candies

#### 2.7.1. Preparation

Two solutions were prepared as follows: (1) dried and powdered Gp-2c (20 mg) was dissolved in 20 mL of aqueous lemon juice (25%, *v*/*v*); (2) 30 mL of water containing agar (1 g) and fructose (7 g). The two solutions were then combined and heated to 80 °C with stirring to obtain a homogeneous mixture. The hot liquid was subsequently dripped, dropwise, into a bath of cold vegetable oil to induce instantaneous gelation and formation of spherical jellies via thermal shock. This method ensured uniform droplet size and rapid stabilization of the gel structure. The jelly spheres were finally collected from the oil bath using a strainer, rinsed three times with water to remove adhering oil, and gently wiped to ensure the surface was clean and oil-free. The medium weight of a sphere was equal to 180 ± 21 mg, based on six measurements. The medium diameter was 4 ± 0.2 mm, measured with a digital caliper.

#### 2.7.2. Color Measurement

The color properties of the gummy jellies were explored by a Chroma Meter CR-5 colorimeter (Konica Minolta, Tokyo, Japan), and the CIELab coordinates (L*, a*, and b*) were measured. Then, C* and h° parameters were automatically calculated. Data were expressed as mean ± SD from three measurements from different sites on the sample.

#### 2.7.3. Bioactive Compound Release and Antiradical Capacity

To determine the bioactive release from the jelly candies, they were placed in a hydroalcoholic solution (H_2_O: EtOH, 1:1 *v*/*v*) in a ratio of 1:1 *w*/*v*, and UV-Vis spectra were acquired at 0, 2, 5, 10, and 15 min. The data acquired were corroborated by HPLC-UV-DAD analysis. To this end, a calibration curve was constructed in the range of 0.0125–0.5 mg/mL and injected under the same conditions previously detailed (Section 2.4).

The antiradical capacity of the jellies was evaluated by performing DPPH and ABTS tests, as previously described (Section 2.5).

#### 2.7.4. Bioaccessibility Evaluation

Bioactive compound bioaccessibility was evaluated through simulated in vitro digestion, performing the static model proposed by the COST Action INFOGEST network [16]. To this purpose, at first, the simulated salivary, gastric, and intestinal fluids (SSF, SGF, and SIF, respectively) were prepared as specified by the protocol by mixing saline solutions constituted by the same constituents but in different proportions and were heated at 37 °C before use. 5 g of the jelly candies were considered, as suggested. In the oral phase, SSF (4 mL) was added together with 25 µL of 0.3 M calcium chloride solution and 975 µL of distilled water. The salivary α-amylase enzyme was not considered, as there was no starch in the jelly formulation. Then, NaOH solution (1 M) was added to the mixture to reach a pH value of 7, and after that, the sample was incubated at 37 °C for 2 min. At the end of this phase, the oral bolus underwent simulated digestion in the gastric environment, adding 7.5 mL of SGF and 1.6 mL of pepsin solution (2000 U/mL, final concentration prepared in SGF), 5 µL of 0.3 M calcium chloride solution, 200 µL of 6 M HCl (to decrease the pH value to 3), and water to reach a total volume of 10 mL. The obtained sample was stirred at 37 °C for 2 h. Finally, to reproduce the intestinal digestion phase, the gastric chyme was mixed with 11 mL of SIF, 5 mL of pancreatin solution (100 U/mL, final concentration prepared in SIF), 2.5 mL of bile salts (10 mM final concentration), 40 µL of 0.3 M calcium chloride, NaOH to increase again the pH value, and water just enough to reach a 1:1 (*v*/*v*) ratio with the gastric chyme. Incubation was performed at 37 °C for 2 h under stirring. At the end of the gastric and intestinal phases, liquid nitrogen was employed to stop any enzymatic activity. Before further chemical analysis, the samples were centrifuged at 4800 rpm for 10 min using a Beckman GS-15R centrifuge (Beckman Coulter, Milano, Italy) equipped with an S4180 rotor. The supernatants were investigated through HPLC-UV-DAD analysis to quantify the amount of Gp-2c released.

#### 2.7.5. Consumer Perception: Sensory Panel Test

A sensory evaluation was conducted by a survey (Figure S2) using 116 non-trained panelists (34 males, 81 females, 1 not declared, aged > 18) and comprised two parts: (1) generic questions about age, sex, job, and knowledge of nutraceutical products and previous use of food supplements; (2) a descriptive analysis to characterize the sensory profile of the jelly candies, using a ranking test to assess consumer preferences. In particular, participants rated the intensity of specific attributes using a four-point Likert scale, where 1 indicated the lowest and 4 the highest score. Evaluated parameters included: appearance, color, shape, texture, chewiness, oral melting, flavor, aftertaste, and overall satisfaction. Finally, panelists were asked to provide suggestions and/or comments, if any. The panelists were led through all the steps of the evaluation thanks to the instructions provided. All participants were informed about the aim and procedures of the study and gave their written informed consent before participation (Figure S3). Participation was voluntary, and subjects received no compensation and could withdraw at any time. Ethical permission was not required for this study, as the sensory panel test on candies concerns only the evaluation of product quality and sensory attributes (such as taste, texture, appearance, and aroma). This type of activity is classified as a sensory and consumer test and does not involve any medical, pharmacological, or invasive procedures on human subjects. Therefore, it does not fall within the scope of studies requiring ethical approval under Italian regulations. In particular, according to Italian Legislative Decree No. 211/2003, which implements Directive 2001/20/EC on clinical trials of medicinal products for human use, and subsequent national guidelines, only studies involving medical or physiological interventions on participants require the evaluation of an Ethics Committee. All data were collected and processed anonymously, in compliance with the EU General Data Protection Regulation (GDPR 2016/679).

Data collected were analyzed through multivariate analysis performed by OriginPro 2015 software (OriginLab Corp., Northampton, MA, USA).

## 3. Results and Discussion

### 3.1. Preface to the Experimental Setup

The wine-producing companies in the Campania region (Italy) were contacted via email and/or social media platforms to invite them for an interview, aimed at gathering data on the quantities and actual uses of by-products and waste generated along the regional winemaking supply chain, and to identify the main sectors in which such materials have been currently valorized. The survey was composed by questions covering key thematic areas relevant to the study: general company information (province, company size), estimated amounts of by-products or waste produced per hectare, with a focus on grape pomace, stalks, and pruning residues, and the respective fields of application (agronomic, zootechnical, industrial, and energy-related, with an additional option for distillation purposes specifically in the case of grape pomace). Furthermore, companies were asked whether these by-products and wastes were managed internally or outsourced to external service providers. This allowed for the assessment of the degree to which circular economy principles are being implemented, that is, the extent to which residues are reused to generate value-added products and/or energy, thereby contributing to economic and environmental sustainability. A total of 84 responses were collected anonymously and processed quantitatively to provide an overall picture of the regional situation (Figure S4).

About 87% of the vineyards were small-scale (1–5 ha and 5–20 ha) in terms of cultivated area. Considering the medium amount of generated wastes and by-products, accounting for 5–10 q/ha, the grape pomace contributed about 40%, whereas the maximum yield (>100 q/ha) contributed about 6%. While the collected data represent only a partial snapshot of the winemaking sector in Campania, they nonetheless provide valuable insights and discussion points regarding current practices in by-product and waste management. Indeed, if stalks and pruning residue are primarily reused on-site for agronomic purposes (e.g., as soil fertilizer), most of the produced grape pomace (about 63%) is sent to external companies for distillation.

In light of the data collected, it is clear that in the Campania region, the exploitation of grape pomace in the food, cosmetic, and pharmaceutical industries (e.g., as a source of bioactive compounds such as polyphenols) is still very limited. This awareness was the driving force behind the proposal of a new reuse for grape pomace, which could address the needs of food sustainability.

### 3.2. From Raw Grape Pomace to Purified Bioactives: Chemical and Nutraceutical Insights

**Figure 1:**
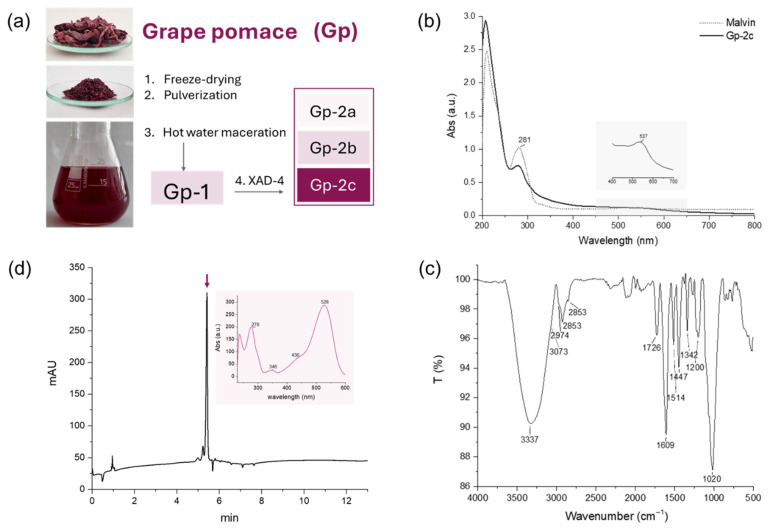
(**a**) Extraction and fractionation workflow to obtain the investigated sample, named Gp-2c, and its (**b**) UV-VIS and (**c**) ATR FTIR spectra, and (**d**) HPLC-UV-DAD chromatogram recorded at 525 nm and the UV-DAD spectrum referred to the peak highlighted with the arrow.

After collection from a wine-producing company, the grape pomace was freeze-dried, which led to the removal of 79% of water, then pulverized and subjected to maceration in hot water at 70 °C. The main consideration in choosing this approach was to favor the extraction of polyphenols, thanks to temperature, but without exceeding higher values that could lead to artifacts due to compound degradation. This low-cost and environmentally friendly protocol could be of interest for a future scale-up in industrial applications. Gel Permeation Chromatography (GPC) of the aqueous extract on Amberlite XAD-4 resin led to a fraction (hereafter named Gp-2c) deprived of simple sugars and other primary metabolites that did not fall into the scope of the present work (Figure 1a).

#### Spectroscopic and Mass Spectrometric Characterization

Gp-2c underwent at first spectroscopic analysis by UV-Vis and ATR-FTIR to obtain a first insight into its chemical constituents. The UV-Vis spectrum recorded in MeOH, depicted in Figure 1b, showed the typical absorption of polyphenols at 281 nm, which could be ascribed to hydroxybenzoates, stilbenes, flavan-3-ols, or anthocyanins, considering their main chemical skeletons in grapes and related products [17]. Moreover, the presence of a peak at 537 nm was consistent with the occurrence of anthocyanins. Indeed, anthocyanins display a characteristic UV-Vis absorption pattern due to their extended system of eight conjugated double bonds and the positive charge localized on the oxygen atom of the heterocyclic ring under acidic conditions. In particular, the specific detected wavelength in the visible region was in accordance with the malvidin aglycone [18]. In this awareness, the UV-Vis spectrum of Gp-2c was compared to that recorded for malvin (malvidin 3,5-diglucoside) available in the laboratory, showing an almost full superimposition. A further confirmation derived from the ATR FTIR spectrum (Figure 1c), which showed in the fingerprint region the bands at 1609 and 1514 cm^−1^ ascribable to the aromatic C=C stretching vibrations, consistent with the anthocyanin aromatic ring system, together with the C=O stretching band at 1726 cm^−1^, likely due to flavylium cation, and the C–O stretching in the aromatic ring at 1200 cm^−1^. The strong peak at 1020 cm^−1^, associated with C–O–C stretching vibrations, was attributed to the *O*-glycosidic bond. Moreover, the broad band at 3337 cm^−1^ was due to O–H stretching vibrations of phenolic (aglycone skeleton) and alcoholic (sugar ring) hydroxyl groups [19].

The preliminary spectroscopic analysis, although useful to obtain insight into the main class of metabolites, did not provide information about the chemical complexity of the sample. Thus, HPLC-UV-DAD analysis was performed. The chromatogram recorded at 525 nm, depicted in Figure 1d, revealed one main peak eluting at 5.4 min, representing 94% of the Gp-2c fraction based on peak area. This highlights the efficiency of the one-step fractionation method for purification purposes. In fact, no other peaks were detected at the other two set wavelengths (280 and 325 nm). The UV-DAD spectrum confirmed the presence of a glycosylated anthocyanin. Indeed, the elution with acidified (1% formic acid) solvents enhanced the extinction coefficient of the band in the visible region ascribed to flavylium cation, causing at the same time a blue shift effect due to the pH decrease. Moreover, the shoulder detected at 436 nm, whose intensity was about 29% of the λ_max_, confirmed the occurrence of glycosylation, and, in particular, suggested the presence of only one sugar moiety that seemed not acylated, considering that the band at 346 nm was only a small hump [20].

**Figure 2:**
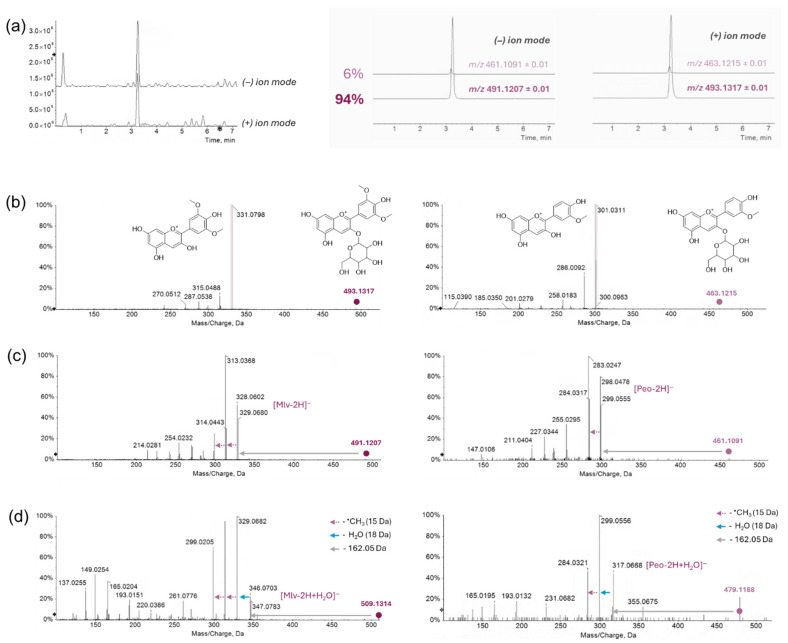
(**a**) Total Ion Current (TIC) chromatograms recorded in negative and positive ion modes and the corresponding eXtracted Ion Chromatograms (XICs) in the gray panel. (**b**) HR-MS/MS spectra acquired in negative ion mode, related to the species [M-2H]^−^. (**c**) HR-MS/MS spectra acquired in negative ion mode, related to the species [M-2H+H_2_O]^−^. (**d**) HR-MS/MS spectra acquired in positive ion mode. Mlv = Malvidin, Peo = Peonidin.

The 94% purity of the main constituent in Gp-2c was further confirmed by UHPLC-HRMS tools (Figure 2a), which also provided pivotal evidence for the structural characterization in negative and positive ESI ion modes (Figure 2b–d)). In particular, based on HR-MS/MS spectra, the main constituent was identified as malvidin 3-*O*-hexoside (e.g., 3-*O*-glucoside, also named oenin), whereas the remaining 6% was ascribed to peonidin 3-*O*-hexoside. Indeed, it was previously reported that in red grapes the hexose moiety linked to monomeric anthocyanins is glucose, and that malvidin 3-*O*-glucoside and its derivatives are typically the predominant monomeric compounds, also contributing significantly to the red coloration of very young red wines, accounting for more than 90% in some red grape varieties [21]. In the positive ion mode, the malvidin and peonidin glycosides were found at *m*/*z* 493.1317 and 463.1215, corresponding to the molecular formulas C_23_H_25_O_12_^+^ (mass error −4.9 ppm) and C_22_H_23_O_11_^+^ (mass error −4.3 ppm), respectively. In both cases, the main neutral loss was related to the sugar moiety (−162.05 Da), giving rise to the corresponding positively charged aglycones at *m*/*z* 331.0798 and 301.0311 (Figure 2b).

**Figure 3:**
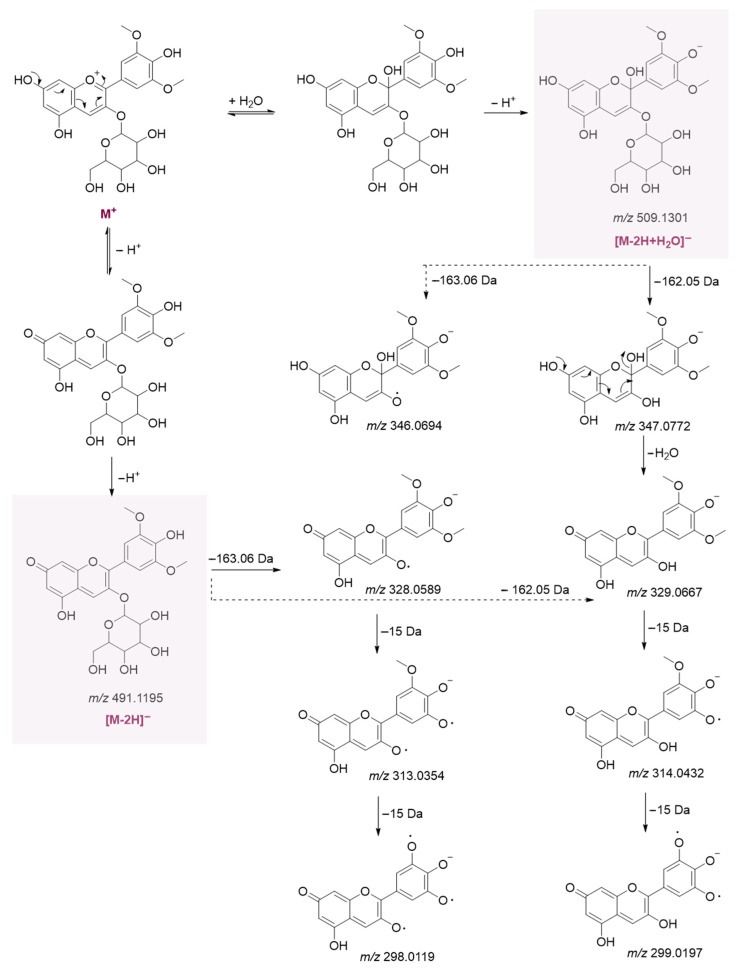
Hypothesized formation and fragmentation of malvidin 3-*O*-hexoside in negative ESI. Theoretical *m*/*z* values are reported below each chemical structure.

In the negative ion mode, the anthocyanins under study were detected as [M-2H]^−^ (at *m*/*z* 491.1207 and 461.1091) and [M-2H+H_2_O]^−^ (at *m*/*z* 509.1314 and 479.1188) ions, whose formation and fragmentation were described according to the literature [22]. In Figure 3, the whole pathway is depicted for malvidin 3-*O*-hexoside.

### 3.3. Gp-2c as the Bioactive Ingredient of Functional Jelly Candies

Anthocyanins are authorized as food additives (E 163) in the EU and have been previously evaluated by JECFA in 1982 and the Scientific Committee on Food (SCF) in 1975. JECFA has established an ADI of 2.5 mg/kg bw/day for anthocyanins from grape skin, while the SCF has not derived an ADI for anthocyanins [23]. This regulatory evidence further supports the suitability of anthocyanin-based formulations for food and nutraceutical applications. In this scenario, the use and effectiveness of bioactive polyphenol compounds could be strongly limited by their poor bioavailability after oral intake, which may depend upon modifications in the gastrointestinal environment and first-pass metabolism reactions. Thus, to overcome these limitations for Gp-2c, it seemed necessary to optimize a formulation, designed for oral administration, able to preserve the nutraceutical functionality of malvidin hexoside, whilst protecting it from degradation, enhancing, sustaining, and controlling release and accessibility. A simple but effective strategy was applied, embedding it into a natural polymer matrix to formulate supplement-like jelly candies. To this purpose, the spherification technique was used, taking advantage of the thermal gelation of agar, a polysaccharide extracted from red algae, able to form a thermo-reversible gel that melts at 85 °C and solidifies at temperatures of 35–40 °C or lower. This strategy led to the obtainment of soft spheres on the inside and solid on the outside. Considering that the chemical structure of the anthocyanin in the form of flavylium cation is pH-dependent, lemon juice was added during the candy manufacturing to maintain the acidic environment necessary to preserve compound stability. Moreover, it acted as an acidulant agent to enhance the jelly flavor and freshness, and also to mask putative aftertastes, thus making it more pleasant to meet the consumers’ compliance. Finally, the preservative effect against microbial growth should be discarded, as it can contribute to the product shelf life. On the other hand, fructose was also added to improve the taste, counteracting the excessive bitterness of lemon juice, to enhance the absorption of the bioactive compound, and to contribute to the texture with a technological function during the candy production process.

Once formulated, the jelly candies underwent color measurement by using the CIELab parameters, defined by the International Commission on Illumination (CIE) in 1976 as a three-dimensional color space used to precisely and uniformly describe color as perceived by the human eye [24]. It is widely used in food, cosmetic, textile, and industrial fields to measure and compare colors. In the CIELAB color space, the color description is based on three coordinates. L* is referred to as lightness, that is, it represents the brightness of the color, ranging from 0 (black) to 100 (white). The recorded value of 48.42 ± 0.5 is in line with a medium bright color (neither very light nor very dark). a* indicates the position of the color along the green-red axis: negative values are in line with a tendency toward green, whereas positive values indicate a tendency toward red. Finally, b* denotes the position of the color along the blue (negative values)-yellow (positive values) axis [25]. Both parameters were positive, showing a strong red component (24.26 ± 0.7) and a slight yellow tendency (4.54 ± 0.5). Taking into consideration a* and b* values, C* (Chroma) and h° (hue angle) parameters were calculated as 24.68 ± 0.8 and 10.59 ± 0.8, respectively. The first represents the color saturation or intensity. In this case, the color can be described as fairly vivid (not dull, not overly intense). The second is measured in degrees (0° = red, 90° = yellow, 180° = green, 270° = blue). Thus, the obtained value is very close to pure red, with a slight orange tint.

#### 3.3.1. Bioactive Compound Release from Jelly Candies and Antiradical Capacity

**Figure 4:**
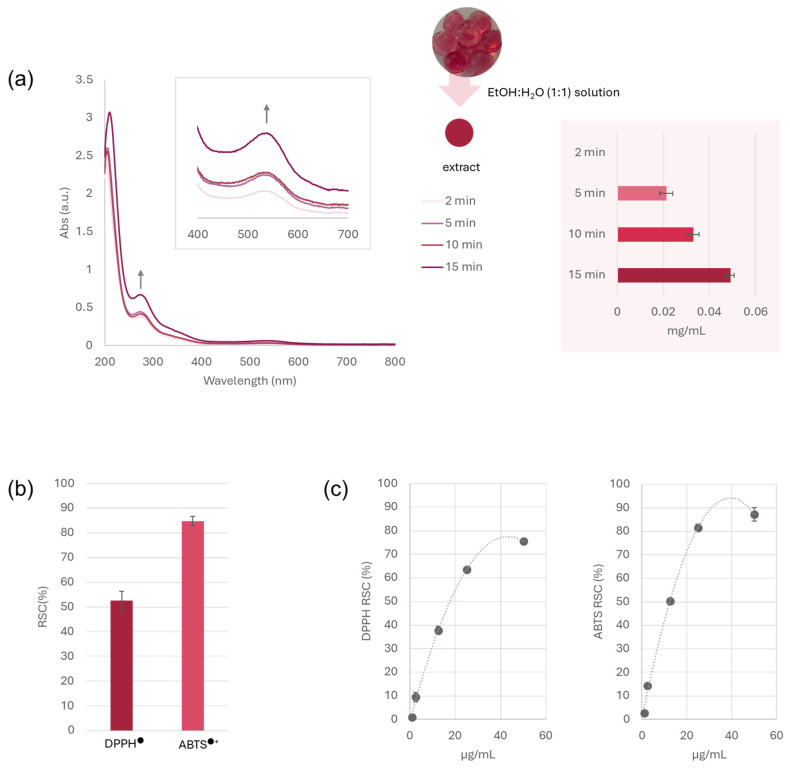
(**a**) Gp-2c release from jellies in EtOH:H_2_O solution, evaluated by UV-Vis spectroscopy and HPLC-UV-DAD (pink box) after 2, 5, 10, and 15 min. (**b**) Antiradical capacity of Gp-2c-based jellies towards DPPH and ABTS^+^ radicals, and of (**c**) Gp-2c solution at 1, 2.5, 12.5, 25, and 50 µg/mL final dose levels.

The release of Gp-2c from jelly candies was evaluated at different times (2, 5, 10, and 15 min) in a hydroalcoholic solution, firstly by UV-Vis spectroscopy. As shown in Figure 4a, the increasing absorbance over time, monitored at the wavelengths of maximum absorption (280 and 538 nm), was in accordance with a progressive release into the medium. However, to quantify more accurately the amount of malvidin 3-*O*-hexoside available to exert the functional effect, the HPLC-UV-DAD analysis was performed, and peak areas were plotted against the pure compound calibration curve at the same time frames (Figure 4a, pink box). Results confirmed the previous observations, suggesting a time-dependent release, which reached its maximum value at 15 min (0.05 mg/mL).

When the jelly candies antiradical capacity was assessed towards DPPH and ABTS^+^ radicals, they showed a differential response depending on the radical species involved (Figure 4b), which followed the behavior of the pure molecule tested in the same condition. This latter demonstrated a dose-dependent activity (Figure 4c), highlighting its antioxidant potential, providing IC_50_ values equal to 17.6 and 12.3 μg/mL in DPPH and ABTS tests, respectively. While the phenolic groups are considered essential for the radical scavenging activity of malvidin 3-*O*-hexoside, it is likely that other factors, such as its conjugated structure, also play a significant role in enhancing its antioxidant properties [26]. Thus, the agar polysaccharide environment in the jelly candies did not seem to mask the interaction with free radicals. In particular, considering the release data previously acquired, the scavenging activity against ABTS^•+^ was perfectly in line with the anthocyanin highest tested dose, confirming the potential use of jelly candies in a nutraceutical scenario. On the contrary, as regards the DPPH test, the scavenging capacity of the anthocyanin in methanol solution at the highest tested dose was higher than that calculated for jellies. It could be an artifact due to the color of the pure molecule solution at 50 µg/mL that could interfere with the spectrophotometric reading at 520 nm, thus being responsible for a higher absorbance value [27]. This drawback should always be taken into account when working with red-colored molecules.

#### 3.3.2. Bioaccessibility of Gp-2c from Jelly Candies by In Vitro Digestion

The antioxidant efficacy of the jelly candies under study represents only a partial view of their promising biological properties. In fact, bioactivity must be evaluated in light of bioaccessibility, as the latter determines the fraction of a compound available to exert physiological effects, by considering the extent to which it withstands digestion [28]. In this context, the use of the standardized INFOGEST protocol [16] provided a robust tool to evaluate the fate of Gp-2c entrapped in the jelly candies during digestion.

**Figure 5:**
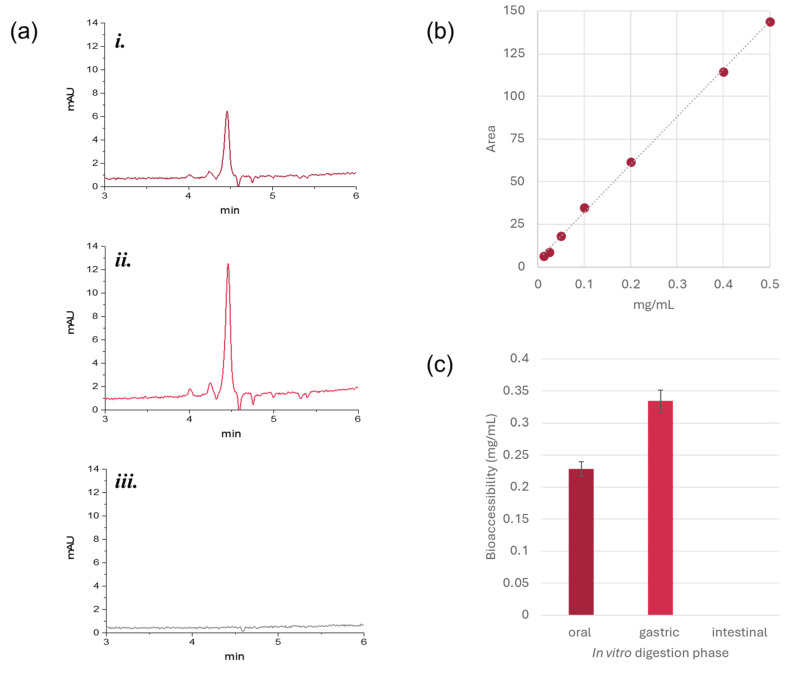
(**a**) HPLC-UV-DAD chromatograms—recorded at 525 nm—of digesta at the end of the three phases of in vitro simulated digestion: (**i**). oral, (**ii**). gastric, (**iii**). intestinal. (**b**) Calibration curve of malvidin 3-*O*-hexoside. (**c**) Bioaccessibility of malvidin 3-*O*-hexoside.

Bioaccessibility data of malvidin 3-*O*-hexoside, estimated by HPLC-UV-DAD at the end of each phase of simulated in vitro digestion, are reported in Figure 5.

The observed trend reflected above all its pH-dependent stability. Indeed, in the oral phase, a moderate amount of the anthocyanin was released from the jelly candies under study, equal to 0.22 ± 0.01 mg/mL, corresponding to about 37% of the initial estimated content (0.6 mg/mL). This is likely due to a partial dissolution of the jelly matrix that began to hydrate and soften in contact with simulated salivary fluids. However, the presence of lemon juice in the jelly composition helped to maintain a mildly acidic medium, thus stabilizing the malvidin flavylium cation form. In the gastric phase the compound highest amount was detected (0.33 ± 0.02 mg/mL, 55% of the initial concentration), suggesting that the strong acidic conditions of this district, together with the presence of the enzymes, disrupt the jelly matrix, leading to improved diffusion of malvidin from the agar network. The structure of the released anthocyanin remains stable, and the amount could be quantified with good accuracy. UHPLC-HRMS analysis confirmed the glycosylated anthocyanin structure integrity. Finally, in the intestinal phase, no traces of the metabolite were revealed, likely due to its estimated very small amount surviving the gastric phase (about 8%), joint with its instability at alkaline pH, leading to rapid structural transformations into chalcones, or other degradation products, including benzoic acid or aldehyde derivatives [29,30]. The presence of bile salts and pancreatic enzymes may also facilitate chemical breakdown or complexation. Thus, the simulated digestion offered key insights for future perspectives. Indeed, it dictated the need to develop a further advanced strategy, able to preserve anthocyanin integrity throughout the entire gastrointestinal tract until intestinal digestion, aimed at enhancing its bioaccessibility for absorption (e.g., the use of pH-responsive delivery systems, co-formulation with stabilizing agents).

### 3.4. Gp-2c-Based Jelly Candies Consumer Perception: Sensory Panel Test

Although the development of a nutraceutical formulation offers a considerable potential for health promotion, its success on the market depends on consumer acceptance. Sensory characteristics (e.g., color, flavor, texture, and overall palatability) play a key role in shaping consumer perception and purchase intent, particularly in confectionery products like jelly candies, where sensory appeal is a primary driver of consumption. To assess the acceptability and perception of jelly candies enriched with Gp-2c a sensory panel test was performed through a structured survey involving 116 non-trained panelists. The choice to recruit untrained participants was intentional, aiming to simulate real-world consumer behavior and preferences, and to reflect broader population-level trends rather than expert-driven judgments. The survey was divided into two sections. Although the questionnaire was anonymous, in the first section, some information, including age, gender, and occupation, was asked, and also the participants’ awareness of nutraceutical products and their previous use of food supplements, in order to obtain a proper context for interpreting the recorded preference patterns. These data are summarized in Figure S5. Briefly, most of the participants were young adults (aged < 30) and predominantly students, reflecting a population that is both open to innovation in nutraceuticals and likely to be influenced by sensory appeal. The clear predominance of female gender (70%) may also have influenced certain sensory preferences, as previous studies suggest gender-based differences in sweetness perception and texture preferences [31,32]. Interestingly, although about 90% of recruited people reported at least some awareness of nutraceuticals, only 36% declared a regular (monthly or more) use of jelly candies, and 57% had never used dietary supplements in gummy form. This indicates that, while the concept of functional foods is not unfamiliar, gummy-based delivery formats still represent a relatively novel vehicle for many consumers in this age group. Such findings reinforce the need to ensure that functional innovations are matched by a strong sensory appeal to facilitate consumer acceptance.

**Figure 6:**
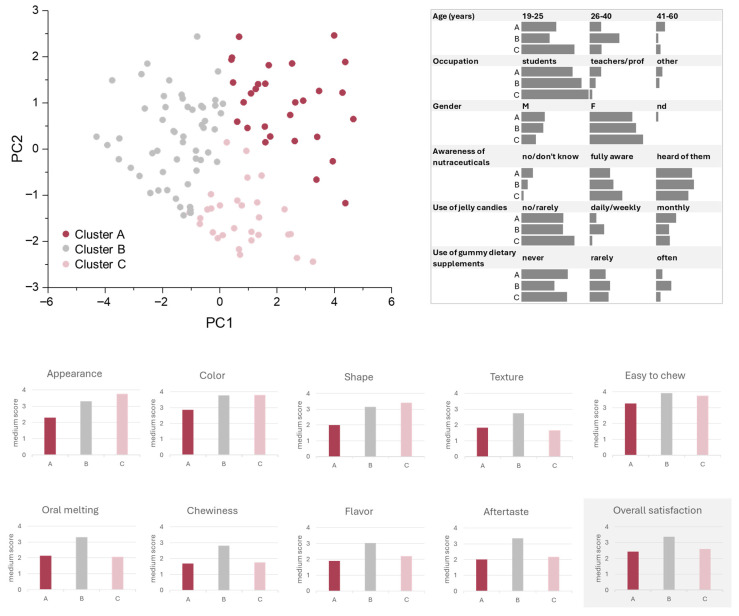
Principal component analysis (PCA) and k-means clustering (k = 3) of sensory evaluation data from 116 untrained panelists assessing Gp-2c–enriched jelly candies, and their sociodemographic and behavioral profiles. The median scores (4-point Likert scale; 1 = lowest, 4 = highest) for sensory attributes are also reported, including appearance, color, shape, texture, chewiness, oral melting, flavor, aftertaste, and overall satisfaction.

The second part of the survey focused on a descriptive sensory analysis to characterize the jelly candies organoleptic profile. Using a four-point Likert scale (1 = lowest; 4 = highest), participants evaluated specific attributes, such as appearance, color, shape, texture, chewiness, oral melting, flavor, aftertaste, and overall satisfaction. The dataset obtained from the responses to the survey was analyzed by principal component analysis (PCA) combined with k-means clustering (k = 3). As depicted in Figure 6, the heterogeneity in panelists’ perception was reduced while preserving key variance. In particular, sensory preferences were clustered in three distinct groups (named cluster A, B, and C). Furthermore, to obtain a deeper correlation between the sensory evaluation and sociodemographic insights collected in the first part of the survey, the results were also analyzed in terms of gender distribution, age, occupation, and familiarity with dietary supplements and jelly products. Cluster B showed high median scores for most of the attributes, among which were texture, oral melting, chewiness, aftertaste, and flavor. This observation is in line with a high overall acceptability of the proposed product, also confirmed by the highest overall satisfaction score among all groups.

The group was more heterogeneous in terms of age, with 44% of panelists falling between 26 and 40 years. They declared themselves to be regular consumers of jelly candies, with 21% reporting daily/weekly use. It was also the group most open to dietary supplements in gummy form, including 23% frequent users, and almost 30% of them have tried these products at least occasionally.

On the contrary, cluster A exhibited the lowest median ratings across all attributes, in that the participants belonging to it seemed to find the product less appealing both in terms of structural and organoleptic attributes. The low satisfaction score in this cluster may suggest a discrepancy between jelly features and consumer expectations, likely due to individual differences in texture sensitivity or flavor preferences. Indeed, this group showed the lowest proportion of female participants (63% vs. 68 and 79% of clusters B and C, respectively) and counted the highest number of adults aged between 41 and 60 years. As regards occupation information, it was largely composed of workers (teachers, professors, others), whose different expectations could be related to more conservative taste preferences. It is also worth noting that the limited inclination toward the use of dietary supplements (with 67% having never used them and 23% using them only rarely) could represent a future challenge. Engaging this group may require targeted improvements in flavor and texture, or a stronger emphasis on tangible health benefits.

Finally, panelists grouped in cluster C provided intermediate responses, which were clearly distinguishable for the highest scores regarding appearance, shape, and color, but had moderate to low ratings for texture, flavor, and aftertaste. Thus, they appreciated the visual features of the jellies but were less satisfied with the gustative experience. Moreover, they found the candies easy to chew, although they seemed to dislike the chewiness and the oral melting, to which low values were assigned. It was composed primarily of young, female students. Interestingly, this cluster was composed mostly of panelists, who are used to consuming jelly candies only rarely and who, in over two-thirds of cases, had never used dietary supplements in gummy form. These findings suggest a group more motivated by novelty, format, and visual appeal than by functional use. Thus, it could represent an ideal target for seasonal editions, limited-release designs, or marketing strategies focused on visual identity and experiential appeal.

In light of the above, group B may represent the primary target among future potential consumers of nutraceutical jelly candies, whereas the other two groups may require education or storytelling to embrace the functional purpose beyond taste.

## 4. Conclusions

This study proves the successful re-evaluation of “low-tech” processes in modern food chemistry, applied to grape pomace to obtain a pure anthocyanin functional ingredient, namely malvidin 3-*O*-glucoside. Its embedment into an agar jelly matrix resulted in a formulation with notable antioxidant activity and time-dependent compound release. Despite the loss of anthocyanin integrity in the intestinal phase of the simulated gastric digestion, the data support its functional relevance as a nutraceutical ingredient. Furthermore, the sensory evaluation highlighted good consumer acceptability, especially among younger, supplement-familiar panelists. These findings align with circular economy principles, offering a feasible, scalable approach to the valorization of agri-food residues. Future research will focus on enhancing intestinal stability by exploring advanced delivery systems to fully exploit the potential of grape pomace-derived anthocyanins in food and health applications.

## Data Availability

The original contributions presented in this study are included in the article/Supplementary Material. Further inquiries can be directed to the corresponding author.

## Supplementary Materials

The following supporting information can be downloaded at https://www.mdpi.com/article/10.3390/foods14223967/s1: Figure S1: Survey distributed to wine-producing companies from the Campania region, related to their management of waste and processing residues; Figure S2: Sensory panel test for consumer perception of produced jelly candies: 16-question survey; Figure S3: Informed consent form distributed to the panelists involved in the sensory panel test for consumer perception; Figure S4: (a) Distribution (%) in the five provinces of the Campania region (Italy) and size (ha) of the 84 vineyards that answered the survey, and (b) their declared waste and by-product production (grape pomace, stalks, and pruning residues) per hectare. (c) Main fields of application for a sustainable reuse of wastes and by-products along the regional winemaking supply chain; Figure S5: General data from participants to the survey: (a) gender, (b) age, (c) occupation, (d) awareness of nutraceutical products, (e) previous frequency of use of jelly candies, (f) previous frequency of use of dietary supplements in gummy forms.
