# Supplementary material for: Bioprocessing of Grape Pomace for the Development of a Nutraceutical Formulation: Bridging Winemaking By-Products and Functional Innovation
Source: Foods. 2025 Nov 19;14(22):3967. doi: 10.3390/foods14223967 (PMC12652182; doi:10.3390/foods14223967)
Supplement: Supplementary file 1 [file foods-14-03967-s001.zip › foods-3993876-supplementary.pdf]

Supplementary materials

Bioprocessing of grape pomace for the development of a nutraceutical formulation: bridging winemaking by-products and functional innovation

Simona Piccolella *, Lucia Mucci, Francesca Prato and Severina Pacifico

Utilization of By-Products and Waste

As part of a research project aimed at promoting sustainability and the valorization of by-products from the wine industry, we are collecting information on current practices in the management of waste and processing residues.

Your participation in this short survey (it takes less than 5 minutes) will be essential to help identify opportunities for innovation, waste reduction, and potential future collaborations.

Thank you in advance for your valuable contribution!

How does the survey work?

The survey is anonymous. However, we kindly ask you to first answer a few questions to help us group participants for data analysis.

1. Location (Province – Campania region)

a. Avellino
b. Benevento
c. Caserta
d. Napoli
e. Salerno

2. Vineyard size (expressed in ha)

a. < 1 ha
b. 1-5 ha
c. 5-20 ha
d. 20-50 ha
e. > 50 ha

3. Grape Pomace: approximate amount produced *per* hectare

a. < 10 q
b. 10-50 q
c. 50-100 q
d. > 100 q

4. Stalks: approximate amount produced *per* hectare

a. < 10 q
b. 10-50 q
c. 50-100 q
d. > 100 q

5. Pruning residues: approximate amount produced *per* hectare

a. < 10 q
b. 10-50 q
c. 50-100 q
d. > 100 q

6. Which is the main end use of grape pomace? (Multiple answers can be selected)

□ Distillation (internal)
□ Distillation (outsourced)
□ Agronomic (internal)
□ Agronomic (outsourced)
□ Zootechnical (internal)
□ Zootechnical (outsourced)
□ Industrial (internal)
□ Industrial (outsourced)
□ Energy-related (internal)
□ Energy-related (outsourced)

7. Which is the main end use of stalks? (Multiple answers can be selected)

□ Distillation (internal)
□ Distillation (outsourced)
□ Agronomic (internal)
□ Agronomic (outsourced)
□ Zootechnical (internal)
□ Zootechnical (outsourced)
□ Industrial (internal)
□ Industrial (outsourced)
□ Energy-related (internal)
□ Energy-related (outsourced)

8. Which is the main end use of pruning residues? (Multiple answers can be selected)

□ Distillation (internal)
□ Distillation (outsourced)
□ Agronomic (internal)
□ Agronomic (outsourced)
□ Zootechnical (internal)
□ Zootechnical (outsourced)
□ Industrial (internal)
□ Industrial (outsourced)
□ Energy-related (internal)
□ Energy-related (outsourced)

Figure S1. Survey distributed to wine-producing companies from the Campania region, related to their management of waste and processing residues.

Jelly candies panel test

Thank you for choosing to participate in this panel test!
You are about to taste one or more gummy candies made with agar, a natural gelling agent derived from algae and enriched with anthocyanins extracted from grape pomace – natural compounds responsible for the red-violet color of grapes and berries.

How does the test work?
The test is anonymous. However, we kindly ask you to first answer a few questions to help us group participants for data analysis.

1. You identify as...
•Male
•Female
•Prefer not to say

2. What is you occupation?
•Student
•Teacher/Professor
•Other (please specify) ______________

3. How old are you?
•< 15
•16-18
•19-25
•26-30
•31-40
•41-50
•51-60
•> 60

4. Before this event, were you aware of nutraceutical products?
•Yes, I am fully aware
•Yes, I have heard of them
•No
•I don't know

5. Do you usually eat jelly candies? If yes, how often?
•Yes, daily
•Yes, weekly
•Yes, monthly
•Very rarely
•No

6. Have you ever taken dietary supplements in gummy form?
•Yes, often
•Yes, rarely
•No, never

Now, please follow the instructions before answering the following questions:

1. Observe the candy (appearance, color, shape).
2. Taste it and focus on texture, flavor, and aftertaste.
3. Answer the questions.

There are no right or wrong answers – we're simply interested in your impressions. It will only take a couple of minutes to contribute to research in the nutraceutical field.

7. How would you rate the appearance of the gummies? Rate from 1 (negative) to 4 (very positive)

1	2	3	4

8. Is the color appealing? Rate from 1 (not at all) to 4 (very)

1	2	3	4

9. Is the shape pleasant? Rate from 1 (not at all) to 4 (very)

1	2	3	4

10. How pleasant is the texture? Rate from 1 (not at all) to 4 (very)

1	2	3	4

11. Is the chewiness appropriate? Rate from 1 (not at all) to 4 (very)

1	2	3	4

12. Does the candy melt pleasantly in the mouth? Rate from 1 (not at all) to 4 (very)

1	2	3	4

13. Is the candy easy to chew? Rate from 1 (not at all) to 4 (very)

1	2	3	4

14. Do you like the flavor? Rate from 1 (not at all) to 4 (very)

1	2	3	4

15. Is the aftertaste pleasant? Rate from 1 (not at all) to 4 (very)

1	2	3	4

16. What is your overall level of satisfaction? Rate from 1 (not very positive) to 4 (very positive)

1	2	3	4

Do you have any suggestions or comments about the candies you tasted?

____

Figure S2. Sensory panel test for consumer perception of produced jelly candies: 16-question survey.

INFORMED CONSENT FORM

I, the undersigned____
born in______________________________ on ___/___/______
living in______________________ address _________________________________
Tel.___________________ e-mail ______________________________________

DECLARE THAT

- I am of legal age.
- I have carefully read all sections of the information sheet.
- I have received clear and comprehensive explanations regarding the request to participate in the proposed sensory analysis, as described in the information sheet.
- The nature, purpose, and procedures of the study have been clearly explained to me, and I fully understand them.
- I have had the opportunity to ask any questions and have received satisfactory answers.
- I have been given sufficient time to consider the information provided.
- I have been informed that the results of the sensory analysis may be published or presented to the scientific community, and that my personal identity will remain confidential in accordance with current privacy regulations (EU Regulation 2016/679 – GDPR and Italian Legislative Decree No. 196/2003, as amended).
- I understand that participation is entirely voluntary, and that I may withdraw at any time without providing any reason.
- I understand that refusal to participate or withdrawal from the study will not entail any negative consequences for me.
- I understand that no financial compensation is provided for participation.
- I have received a copy of this informed consent form.

I THEREFORE FREELY DECLARE THAT I

☐ wish ☐ DO NOT wish
to participate in the proposed sensory analysis.

Place, date _______________ Signature _______________

*Name and surname of the person obtaining consent*____

Date _______________ *Signature* ______________________________________

Figure S3. Informed consent form distributed to the panelists involved in the sensory panel test for consumer perception

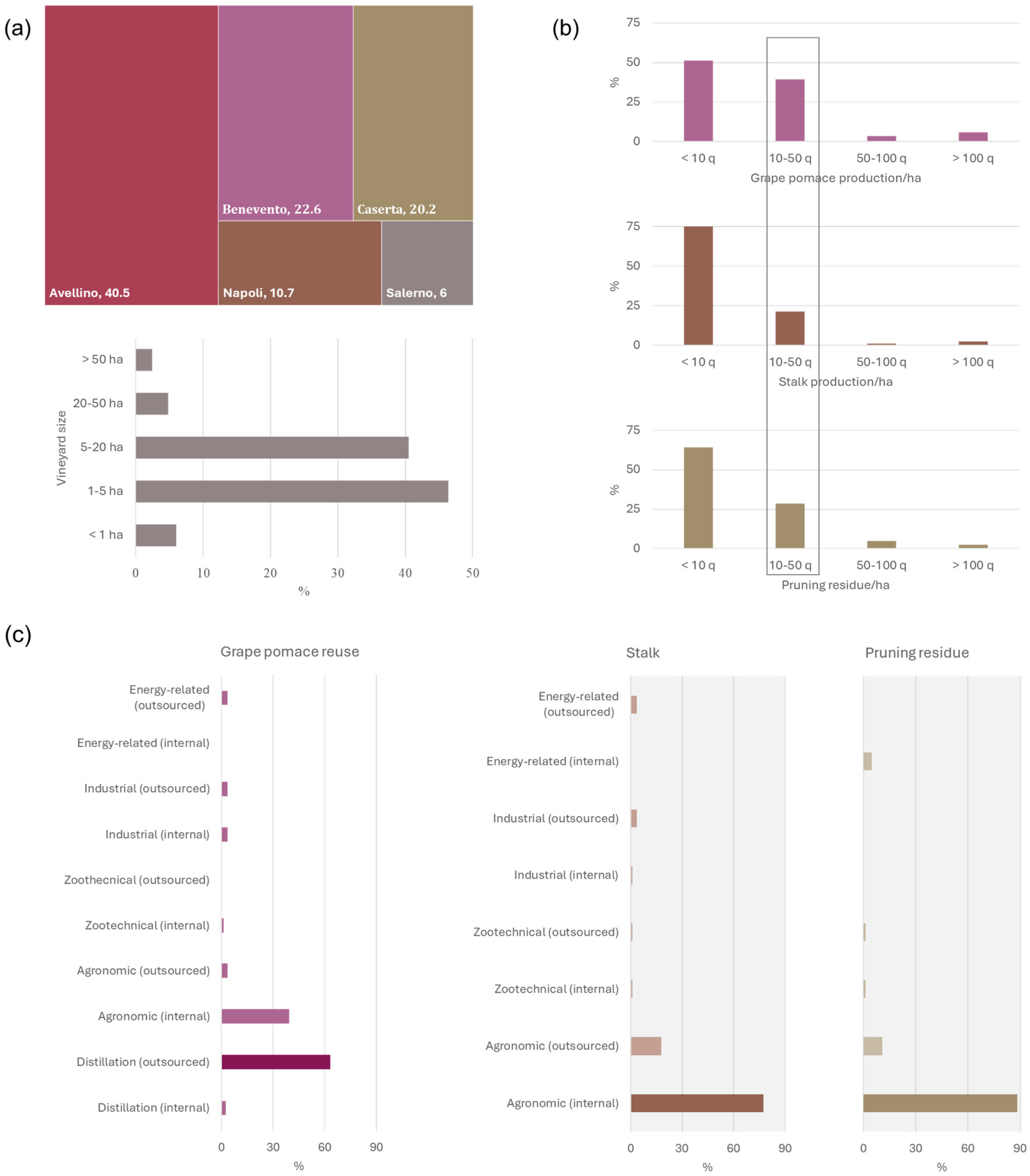

Figure S4. (**a**) Distribution (%) in the five provinces of the Campania region (Italy) and size (ha) of the 84 vineyards that answered the survey, and (**b**) their declared waste and by-product production (grape pomace, stalks, and pruning residues) *per* hectare. (**c**) Main fields of application for a sustainable reuse of wastes and by-products along the regional winemaking supply chain.

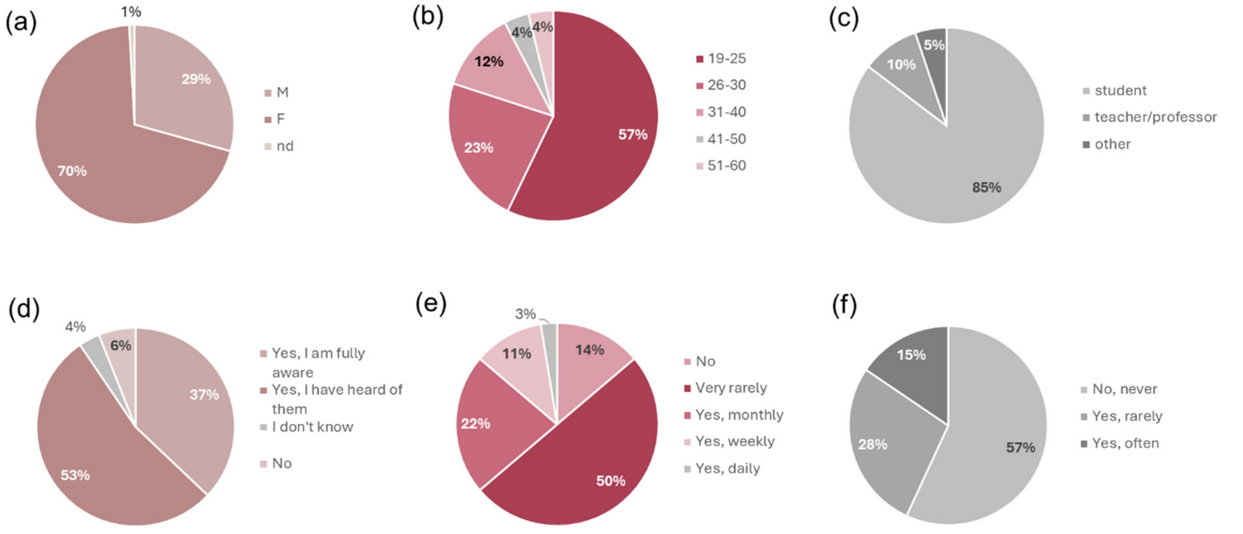

Figure S5. General data from participants to the survey: (**a**) gender, (**b**) age, (**c**) occupation, (**d**) awareness of nutraceutical products, (**e**) previous frequency of use of jelly candies, (**f**) previous frequency of use of dietary supplements in gummy forms.
